# A cost-effective RNA extraction technique from animal cells and tissue using silica columns

**DOI:** 10.14440/jbm.2017.184

**Published:** 2017-05-10

**Authors:** Mario D. Escobar, Jason L. Hunt

**Affiliations:** Biology Department, College of Agriculture and Life Sciences, Brigham Young University Idaho, Rexburg, ID 83460, USA

**Keywords:** cell culture, extraction, RNA, silica, tissue

## Abstract

Ribonucleic acid (RNA) is widely used in molecular biology assays, and some of the most common assays include: northern blotting and RT-PCR gene expression analysis. RNA is generally extracted by two methods: phenol-chloroform or commercially available silica spin column kits. Phenol-chloroform extraction is generally more economical; however, it produces hazardous byproducts, and leftover chemicals in the sample that can inhibit downstream applications. Commercial kits usually have simple set ups and short preparation time; however, they can introduce a significant expense to laboratory budgets. Here we have created a method to extract RNA using generic silica columns and readily available reagents while maintaining a high yield and purity.

## INTRODUCTION

Ribonucleic acid (RNA) is a ribose based nucleic acid that uses guanine, uracil, adenine, and cytosine as nitrogenous bases. RNA is found throughout the cell exhibiting functions that range from a template for protein synthesis [1] to catalyzation of biological reactions [2]. With the increased availability of cost effective and rapid genomic sequencing resources, RNA has proved extremely valuable in the analysis of entire transcriptomes. The isolation and purification of RNA can be complicated because of the presence of ribonuclease enzymes that rapidly degrade RNA. Although there are numerous ways to extract and isolate RNA, most labs gravitate toward using phenol-chloroform extractions or commercially available kits. Phenol-chloroform extractions use the property of phenol to separate the proteins from the nucleic acids, allowing for easy capture of the nucleic acids with ethanol precipitation. Although, this method has become a staple for RNA extraction for many scientists because of its high RNA yield and purity [3], the long hands-on time, the hazardous waste, and sensitive steps make it a very inefficient technique. Commercially available kits cut down on time and hazardous waste, and make use of very simple protocols which reduce the likelihood of mistakes. However, these kits can be very expensive with higher end kits costing upwards of $6.00 per extraction. This high cost can become a significant expense in the small lab’s budget. In our article, we present a method using guanidine thiocyanate, a chaotropic salt with known denaturing abilities [4], to extract RNA and then bind it to low-cost silica columns while maintaining a high yield and quality at an approximate cost of $0.48/sample.

## MATERIALS AND METHODS

### Cell culture

GMMe cells obtained from the American Type Culture Collection (ATCC, Manassas, VA, CRL-267) were grown in Dulbecco’s Modified Eagle Medium/Nutrient Mixture F-12 (DMEM/F12; Caisson Labs, Smithfield, UT, DFL14-500ML) with 10% FBS (Fetal Bovine Serum; Caisson Labs, Smithfield, UT, FBL02-500ML) at 37°C and 5% CO_2_. After 48 h of growth, the cells were trypsinized, counted and prepared for RNA extraction.

### Buffer creation

Buffer composition is summarized in **Table 1**. Buffer A was composed of 4 M guanidine thiocyanate (GITC; Promega, Madison, WI, V2791), 10 mM 2-(N-morpholino)ethanesulfonic acid (MES; Fisher Scientific, Hampton, NH, BP300-100) pH 5.5, and 1% β-mercaptoethanol (β-M; Sigma Aldrich, St. LouisMO, M6250-10ML). β-M was added immediately before starting the extraction. Buffer B was composed of 1 M GITC, 10 mM tris(hydroxymethyl)aminomethane (Tris; Fisher Scientific, Hampton, NH, BP152-1) pH 7. Buffer C was composed of 80% Ethanol (Decon Labs, King of Prussia, PA, V1005HC), and 10 mM Tris buffer pH 7.

### Cell RNA extraction

Cell culture media was carefully removed from flasks (T-160) and the cells were washed in 15 ml phosphate-buffered saline (PBS). Cells were then incubated for 10 min with 4 ml of trypsin (Caisson Labs, Smithfield, UT, TRL02-100ML) and 12 ml of PBS at 37°C, and 5% CO_2_. After incubation, the cell mixture was transferred into a new 50 ml centrifuge tube (VWR, Radnor, PA, 89039-664) and centrifuged at 1600 G for 6 min at 25°C. The supernatant was carefully removed and the subsequent cell pellet was re-suspended with 3 ml of DMEM/F12 with 10% FBS. A 10 µl aliquot sample of cell mixture was then retrieved and mixed with 10 µl of trypan blue (Thermo Fisher, Waltham, MA, 15250-061). The cell-trypan blue mixture was then loaded into a Countess II FL Automated Cell Counter (Thermo Fisher, Waltham, MA, AMQAF1000) and a cell count was calculated. The cell mixture was then aliquoted into new 1.5 ml centrifuge tubes containing approximately 4.5 × 10^5^ cells per tube. Tubes containing cells were then divided into three extraction procedures that consisted of the proposed silica column procedure, an RNeasy mini kit (Qiagen, Hilden, Germany, 74104) and a GenCatch total RNA miniprep kit (Epoch Life Science, Missouri City, TX, 1660050).

Both kit procedures followed the manufacturer’s instructions with no modification and the subsequent RNA obtained was stored at −80°C. For the silica column procedure, cell culture media was carefully removed and the remaining cells were washed in 1 ml PBS. Following the washing and removal of PBS, 300 µl of Buffer A was added to each well with cells. Cells were then incubated with Buffer A for 15 min. After incubation, 300 µl of 70% ethanol was added to each well and the mixture was added to a silica column (Epoch Life Science, Missouri City, TX, 1920-050) and centrifuged (Eppendorf, Hamburg, Germany, 5424) at 8000 G for 30 s. The resultant flow through was then discarded. The column was then washed again by the addition of 600 µl of Buffer B followed by centrifugation at 8000 G for 30 s. The resultant flow through was again discarded. The column was then washed twice with 500 µl of Buffer C followed each time by centrifugation at 8000 G for 30 s and the flow through was discarded. Following the washing steps, the column was centrifuged again for 2 min and then placed in a new 1.5 ml centrifuge tube (VWR, Radnor, PA, 89000-010). The RNA was captured by adding 30 µl of RNase free Mili-Q water to the column followed by centrifugation at 8000 G for 30 s. The solution containing the RNA was treated with DNase (Thermo Fisher, Waltham, MA, EN0525) by adding 1 µl of 10 × reaction buffer and 1 µl of DNase. The reaction volume was then adjusted to 10 µl with RNase-free water and then incubated at 37°C for 30 min after which 1 µl of 50 mM ethylenediaminetetraacetic acid (EDTA) was added. Following the addition of EDTA the reaction was incubated at 65°C for 10 min and then stored at **−**80°C.

### Tissue RNA extraction

**Figure 1.**
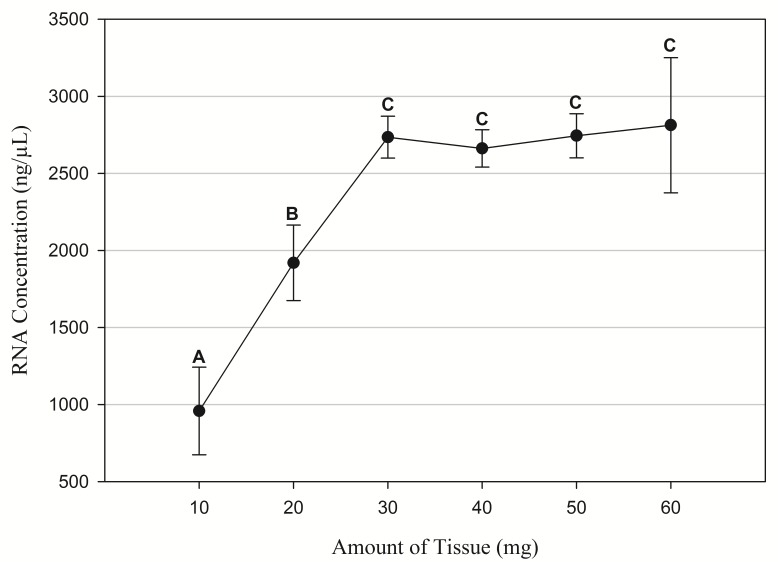
Mean RNA concentration from different initial amounts of tissue. *N* = 9 samples per extraction procedure. Means with different letters (A, B and C) are significant (*P* < 0.05).

Rat (Rattus norvegicus) liver tissue was donated by Clair Eckersell (Brigham Young University - Idaho). Identical to the cell RNA extraction protocols, both kit procedures followed the manufacturer’s instructions using 30 mg of tissue with no modification and the subsequent RNA obtained was stored at **−**80°C. For the silica column procedure, approximately 30 mg of tissue was placed in 2 ml centrifuge tubes (VWR, Radnor, PA, 89000-010) followed by the addition of 600 µl of Buffer A. Tissue was disrupted using a homogenizer (Omni International, Kennesaw, GA, TH115). The resultant mixture was centrifuged at 8000 G for 3 min and the supernatant was transferred to a new 2 ml centrifuge tube and one volume of 70% ethanol was added. The mixture was then added to a silica column and centrifuged at 8000 G for 30 s. The resultant flow through was discarded. The column was then washed with 600 µl of Buffer B followed by centrifugation at 8000 G for 30 s and the flow through was discarded. The column was then washed twice with 500 µl of Buffer C followed each time by centrifugation at 8000 G for 30 s and flow through was discarded. To clear any remaining buffer, the column was centrifuged for 2 min and then placed in a new 1.5 ml centrifuge tube. The RNA was captured by adding 30 µl of RNase free Mili-Q water to the column followed by centrifugation at 8000 G for 30 s. The solution containing the RNA was treated with DNase and then stored at **−**80°C. Additionally, to test the efficacy of the silica column against varying amounts of tissue, we performed incremental extractions from starting tissue samples that ranged from 10 mg to 60 mg (**Fig. 1**).

### Fragment analysis and quantification

RNA was sent to Idaho State University’s Molecular Research Core Facility for the quantification and the fragment analysis. RNA was quantified using an RNA fluorescence assay (Thermo Fisher, Waltham, MA, Q32852) following the manufacturer’s instructions, with a fluorometer (Thermo Fisher, Waltham, MA, Qubit^®^ 2.0), and using spectrometer (Thermo Fisher, Waltham, MA, Nanodrop 1000). RNA fragment was analyzed using a fragment analyzer (Advanced Analytics, Ankeny, IA, Fragment Analyzer) and the results were analyzed by fragment analysis software (Advanced Analytics, Ankeny, IA, PROSize).

## RESULTS AND DISCUSSION

**Figure 2.**
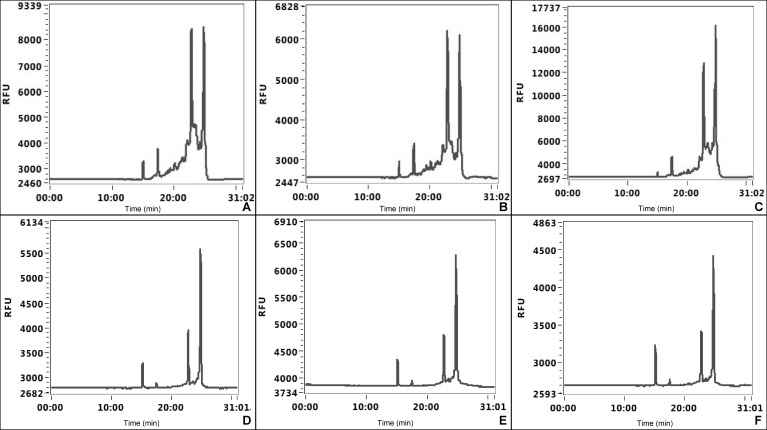
RNA fragment analysis. **A-C.** Tissues samples 1–3. **D-F.** Three independent cell cultures.

Following RNA fragment analysis (Advanced Analytics, Ankeny, IA, PROSize), we found that our high efficiency and low cost extraction technique for RNA from both cells and tissue showed little to no signs of degradation (**Fig. 2**). The RNA fragment analyzer scores RNA integrity with a unit called the RNA quality number (RQN). Although spectroscopy can be used to determine the concentration and purity of RNA it lacks the power to determine the integrity of the RNA. The RQN can estimate the integrity of the RNA using a proprietary algorithm that uses the area before the 18 s peak, the total area of the 18 s and 28 s peaks, and the ratio of the 28 s and 18 s peaks as obtained from the fragment analyzer graph. The RQN has been shown to correlate to the RNA integrity number (RIN) [5]. The RIN numbers range from 0 to 10. The number 0 is the lowest possible value and 10 is the highest possible value. For RNA sequencing, where RNA integrity is essential for significant results, a RIN lower than 6.4 is detrimental to the results [6].

Although the cell samples showed a significantly smaller concentration of RNA in comparison to the tissue (**Table 2**) we hypothesize that this difference in concentrations was the result of the variation in the number of cells obtained from cell culture *vs* the amount of cells used for the procedure or variability in the tissue samples. Our average cell count was around 4 × 105 cells, consistent with the average 12 well plate which has around 4 × 10^5^ cells [7], whereas 30 mg of rat liver has around 4.17 × 10^6^ cells [8]. Additionally, we have observed spectrums of variability of RNA quality and concentration when comparing enzymatically-rich liver tissue to high lipid adipose tissue in commercially available extractions.

All 260/280 ratios for both cells and tissue were around 2.0 indicating that the composition of the eluent was RNA and not DNA or protein carryover. In contrast, the 260/230 ratios in tissue were below 1, indicating a contaminant, possibly a small amount of guanidine thiocyanate salt. However, the cells showed 260/230 ratios above one indicating better purity. We speculate that it would be possible to improve RNA yields by adding chelating agents (like EDTA), anti-foam agents (like isoamyl alcohol) or transcription inhibitors (like sodium lauroyl sarcosinate). However, in order to keep the method simple and cost effective, these reagents were omitted for a later study.

**Figure 3.**
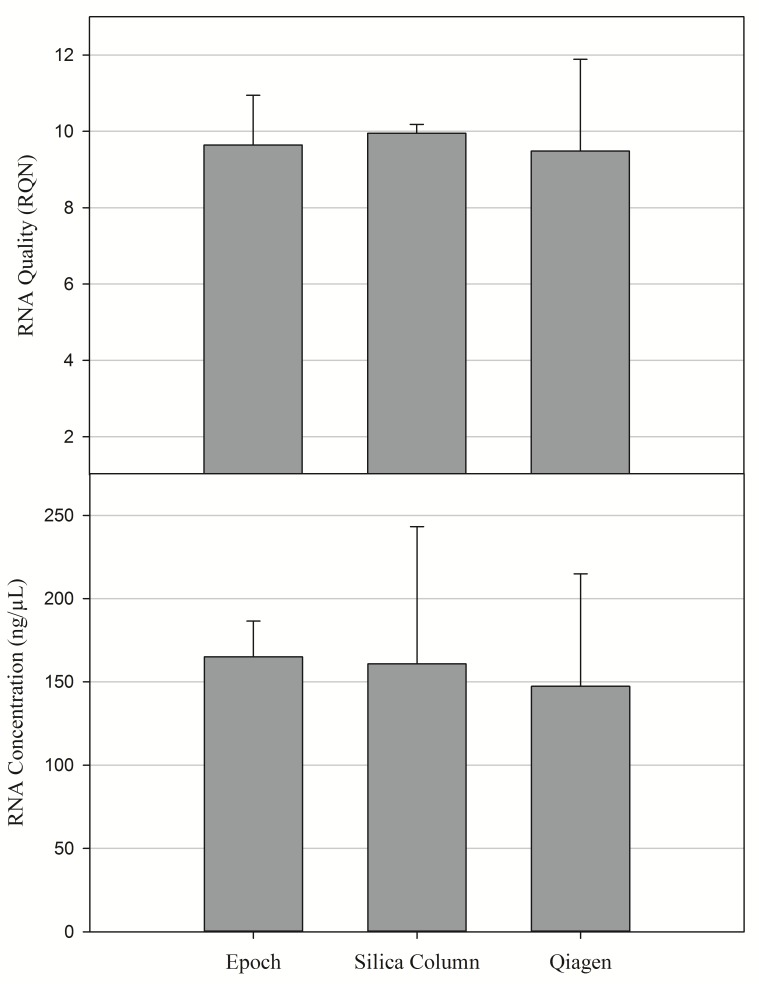
Mean concentration and quality of RNA from different cell extraction procedures. *N* = 12 samples per extraction procedure.

**Figure 4.**
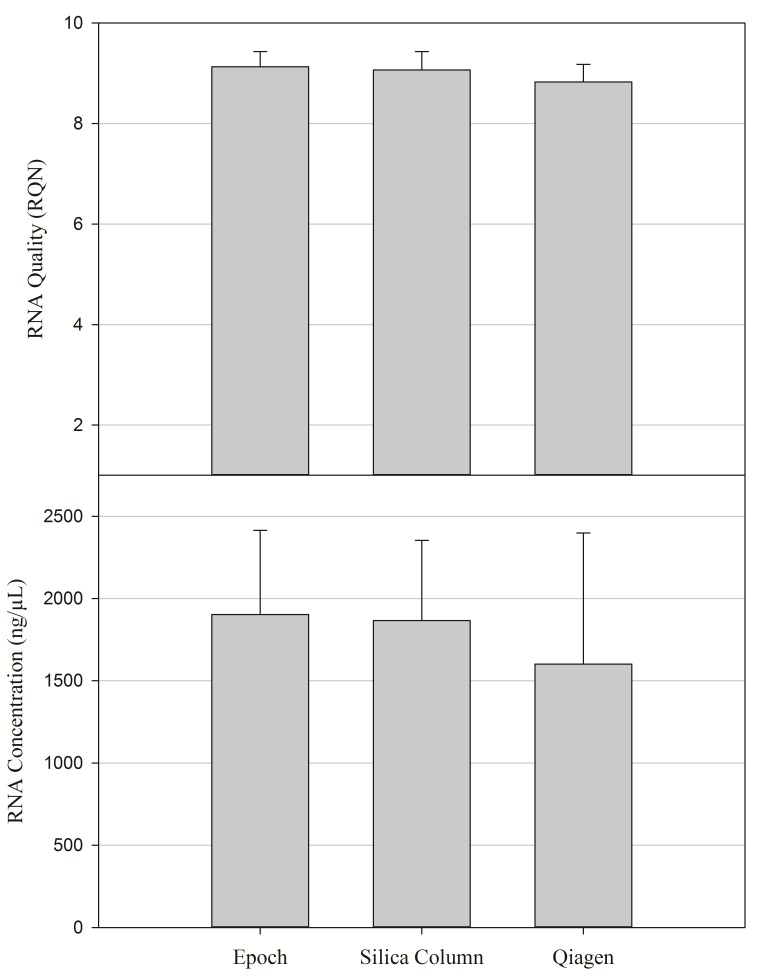
Mean concentration and quality of RNA from different tissue extraction procedures. *N* = 12 samples per extraction procedure.

The RNA results comparing the commercial kits to the silica column procedure showed no significant difference in the amount or quality of RNA (**Fig. 3** and **4**). These results support the claim that the proposed protocol can extract RNA at the same quality and level as the more expensive commercial kits. Although the silica column procedure was effective in extracting pure and consistent RNA, our data suggests that there is a binding limit. After approximately 30 mg of tissue, additional material did not significantly affect the amount of RNA extracted (**Fig. 1**).

Although the idea of binding RNA to free-floating silica particles has been used in the past [9], the complexity and tediousness of using unbounded silica particles, in contrast to using silica columns, can overwhelm the average scientist foreign to the technique. Another possibility is creating in-house silica columns [10]. However, this is such a vital step in the extraction procedure that without proper calibration, standardization, and sterility, large variations in yield and purity are likely to compromise the results. There have been previous attempts to create silica-based extraction protocols that rely on in-house buffers and commercially available columns [11], yet the use of phenol and other hazardous reagents in these protocols can become a problem if not handled properly and could result in injury if the user is still new to the protocol. Although guanidine thiocyanate is minimally hazardous, it is considerably less hazardous than the aforementioned phenol and chloroform.

The procedural time of the protocol with an experienced user is around 20 min, which is comparable to commercially available kits, whereas the preparation of the buffers, which can be done before hand, extended the time by another 15 min. Although a DNase step was added to the procedure to better improve the quality of the RNA, we found that omission of this step only resulted in very small amounts of genomic DNA (unpublished data). With the same omission, we also found small amounts of genomic DNA in commercial kits (unpublished data). Overall, this article aims to aid new laboratories or scientists under tight budget constraints to obtain the same quality of results for RNA isolation as commercially available kits, while using less expensive yet widely available reagents.

## Figures and Tables

**Table 1.** Summary of buffer composition.

Buffer A	4 M	Guanidine thiocyanate
	10 mM	MES pH 5.5
	1%	β-mercaptoethanol
Buffer B	1 M	Guanidine thiocyanate
	10 mM	Tris pH 7
Buffer C	80%	Ethanol
	10 mM	Tris pH 7

**Table 2.** Extracted RNA concentration (ng/µl) and quality assessment (RQN).

Sample ID	Concentration (ng/µl)	260/280	260/230	RQN
Tissue 1	1118.3	2.14	1.36	7.4
Tissue 2	513.14	2.13	0.99	7.3
Tissue 3	746.27	2.11	1.50	8.50
Cells 1	69.52	2.08	1.4	10.00
Cells 2	56.36	2.1	1.29	10.00
Cells 3	55.16	2.18	1.12	10.00
